# Early termination of ISRCTN45828668, a phase 1/2 prospective, randomized study of Sulfasalazine for the treatment of progressing malignant gliomas in adults

**DOI:** 10.1186/1471-2407-9-372

**Published:** 2009-10-19

**Authors:** Pierre A Robe, Didier H Martin, Minh T Nguyen-Khac, Maria Artesi, Manuel Deprez, Adelin Albert, Sophie Vanbelle, Stephane Califice, Markus Bredel, Vincent Bours

**Affiliations:** 1 Department of Surgery (Neurosurgery), University of Liège, Domaine du Sart Tilman, B35, 4000 Liège, Belgium; 2 Department of Medical Statistics, University of Liège, Domaine du Sart Tilman, B35, 4000 Liège, Belgium; 3 Department of Pathology (Neuropathology) University of Liège, Domaine du Sart Tilman, B35, 4000 Liège, Belgium; 4 Department of Neurological Surgery, Northwestern Brain Tumor Institute, Northwestern University, 303 E Superior Street, 60611-3015 Chicago, IL, USA; 5 Department of Human Genetic - CBIG Research Center, University of Liège, Domaine du Sart Tilman, B35, 4000 Liège, Belgium; 6 Oncomethylome Sciences SA, Avenue de l'Hopital 11, 4000 Liège, Belgium; 7 Department of Neurosurgery, University of Utrecht Medical Center, Heidelberglaan 100, 3584 CX Utrecht, the Netherlands

## Abstract

**Background:**

Sulfasalazine, a NF-kappaB and x(c)-cystine/glutamate antiport inhibitor, has demonstrated a strong antitumoral potential in preclinical models of malignant gliomas. As it presents an excellent safety profile, we initiated a phase 1/2 clinical study of this anti-inflammatory drug for the treatment of recurrent WHO grade 3 and 4 astrocytic gliomas in adults.

**Methods:**

10 patients with advanced recurrent anaplastic astrocytoma (n = 2) or glioblastoma (n = 8) aged 32-62 years were recruited prior to the planned interim analysis of the study. Subjects were randomly assigned to daily doses of 1.5, 3, 4.5, or 6 grams of oral sulfasalazine, and treated until clinical or radiological evidence of disease progression or the development of serious or unbearable side effects. Primary endpoints were the evaluation of toxicities according to the CTCAE v.3.0, and the observation of radiological tumor responses based on MacDonald criteria.

**Results:**

No clinical response was observed. One tumor remained stable for 2 months with sulfasalazine treatment, at the lowest daily dose of the drug. The median progression-free survival was 32 days. Side effects were common, as all patients developed grade 1-3 adverse events (mean: 7.2/patient), four patients developed grade 4 toxicity. Two patients died while on treatment or shortly after its discontinuation.

**Conclusion:**

Although the proper influence of sulfasalazine treatment on patient outcome was difficult to ascertain in these debilitated patients with a large tumor burden (median KPS = 50), ISRCTN45828668 was terminated after its interim analysis. This study urges to exert cautiousness in future trials of Sulfasalazine for the treatment of malignant gliomas.

**Trial Registration:**

Current Controlled Trials ISRCTN45828668

## Background

Despite a wealth of preclinical and clinical research, treatment options for malignant gliomas remain scarce, and median survival of patients barely reaches 15 months despite surgery, radiation and chemotherapy [1].

Among several recently discovered biochemical anomalies of malignant gliomas, transcription factor NF-kappaB has emerged as a major molecular determinant of tumor progression and treatment resistance [2]. It is constitutively activated in most high-grade gliomas [3-8], where it promotes the expression of various pro-invasion, anti-apoptotic and cell-cycle related genes [7,9,10]. Inactivation of NF-kappaB blocks glioma growth in *in vitro *and *in vivo *models, and increases their response to radiation, chemotherapy and suicide gene therapy [11-13].

Sulfasalazine, an anti-inflammatory drug, is a known inhibitor of the multimeric kinase complex IKK that is essential to the canonical pathway of NF-kappaB activation [14]. Additionally, this drug inhibits the x(c)-cystine/glutamate antiporter, an amino-acid transporter protein that is essential for the survival of glial cells [15,16]. Although there is presently a lack of pharmacological data that proves sulfasalazine penetrates the blood brain barrier, it was shown to exert a clear anti-tumor effect on rodent and human glioblastomas transplanted in the brain of nude mice [2,12,15].

Because of these promising preclinical data and as Sulfasalazine has been used clinically for over 4 decades for the treatment of colon and joint inflammatory diseases and causes little toxicity at daily doses up to 6 g/day [17], we initiated ISRCTN45828668, a double-blinded, prospective, phase 1/2 clinical trial of Sulfasalazine for the treatment of recurrent or progressive high-grade gliomas [18]. This study was designed as a phase 2, randomized, study looking for tumor responses. Given the fact that no specific safety data was available on the use of Sulfasalazine in the setting of malignant glial tumors however, the study also looked for treatment toxicities as a primary objective, and an interim review of treatment safety was planned to occur after an accrual of 10 patients.

The results of this interim review are reported according to the GNOSIS recommendations in neuro-oncology [19].

## Methods

### Study Design

The protocol of ISRCTN45828668, a prospective, randomized, double-blinded single-center phase 1-2 study of Sulfasalazine for the treatment of recurrent or progressive high-grade astrocytic gliomas, has been published previously [18]. Briefly, a total of twenty adult patients (over 18 years old), with progressive astrocytic malignant glioma despite surgery, radiation therapy and a first line of chemotherapy and with a life expectancy of at least 2 month were scheduled for recruitment. After signing an informed consent, patients underwent a clinical, radiological (baseline MRI) and biological examination and were evaluated with respect to our inclusion and exclusion criteria [see additional file 1: Table S1]. Upon enrollment, subjects were randomly assigned to one of four dose regimens of Sulfasalazine, using a randomization software (S-Plus v6.2, Insightful Corp., MathSoft, Seattle, WA, USA). Continuous daily doses of 1.5, 3, 4, or 6 g were taken orally t.i.d. until complete remission, evidence of progression or drug intolerance. Both the patients and the treating physicians were blinded to the individual dosages of sulfasalazine. Drug preparations were conditioned at the CHU of Liege Pharmacy department, and provided in the form of unremarkable capsules allowing both the patient and the physicians to remain blinded with respect to the individual dosage.

Given that Sulfasalazine safety had never been assessed in the context of malignant glial tumors however, the two primary endpoints of ISRCTN45828668 were the assessment of treatment toxicity and tumor response as measured according to modified (volumetric) MacDonald's criteria [20]. Overall survival and progression-free survival were secondary endpoints. To enforce the assessment of treatment safety, an independent, interim analysis of drug safety was scheduled and conducted after the accrual of ten patients. In order to facilitate the interim analysis of the data by the review committee, the randomization algorithm was weighted so that 8 of the first 10 patients received either the lowest or the highest drug dosage.

The protocol was sponsored by the Department of Neurosurgery, University Hospital of Liège, Belgium. It was reviewed and approved by the Ethics Committee of the Faculty of Medicine of the University of Liège, underwent review and approval by Belgian Federal Health Authorities (authorization reference 548/03/05) and was granted the European Trial database (EudraCT) number 2004-004392-11.

Patients under 18 years old, or with anaplastic oligodendroglioma (WHO grade 3), allergy to sulfa drugs, porphyria, G-6-PD, kidney or liver deficiency or psychiatric disorder deemed incompatible with compliance to the study were excluded, as well as lactating or pregnant women.

Blood samples were analyzed every 15 days following treatment initiation, and follow-up visits-including clinical examination, history, MRI scan and urinalysis - were performed every 30 days. Adverse events were evaluated according to the NCI common toxicity criteria (CTCAE) version 3.0). Volume changes were measured on gadolinium-enhanced T1 weighted MPR images, using the Splicer software v 2.6 (Surgical Planning Laboratory, Brigham and Women's Hospital, Boston, MA [21]).

### Statistical analysis

Results were expressed as means ± standard deviations (SDs) for quantitative variables, while frequencies and proportions (%) were used for categorical variables. The non-parametric correlation coefficient of Spearman was calculated for measuring the association between two quantitative variables. Survival curves were determined by the Kaplan-Meier method and compared by the log-rank test. Calculations were always carried out on the maximum number of data available. Missing data were not replaced. Results were considered to be significant at the 5% critical level (p < 0.05). Data analysis was carried out using SAS (version 9.1 for Windows) and S-PLUS (version 6.2) statistical packages by AA and SV (Department of Medical Statistics, University of Liège, Belgium).

### Immunochemistry and MGMT promoter analysis

Histology of the tumor samples obtained at the time of initial surgery was reviewed in all patients by an experienced neuropathologist (MD). NF-kappaB p65 staining of patient 6's tumor was performed using a monoclonal antibody to p65 (Santa Cruz Biotechnology) and as described previously [2]. MGMT promoter analysis was performed by Oncomethylome Sciences (Liege, Belgium), as described previously [22].

## Results

### Patient demographics

The relevant demographic and clinical characteristics of the patients included in the study are listed in [see additional file 2: Table S2A]. At the time of interim analysis, performed as scheduled, nine males and one female patient, aged 32-62 yr (median: 53.5) had been enrolled in and completed the study between May, 2005 and January, 2007. All patients had undergone surgery, standard radiation therapy (54-60 Gy) and a course of chemotherapy with alkylating agents prior to their inclusion in the present study. Nine patients were thus on temozolomide chemotherapy prior to their inclusion in the study. One patient was on a BCNU regimen. All patients had been off these cytotoxic medications for at least 4 weeks prior to the initiation of their Sulfasalazine treatment, and none had manifested severe side effects. Patient #4 and 6 had however experienced mild headaches and nausea while on temozolomide, and patient #7 had presented transiently decreased white cell counts on this regimen. All patients were free of those symptoms at the time of inclusion in ISRCTN45828668.

Eight patients had a diagnosis of recurrent glioblastoma multiforme and two suffered progressive anaplastic astrocytomas. MGMT promoter methylation analysis, assessed in all tumors at the time of initial resection, provided unequivocal results in 8 patients, and demonstrated a promoter methylation in only one tumor (patient # 3). KPS scores at the time of inclusion ranged from 40 to 70, with a median of 50. The initial tumor volume was on average 67.7 ± 31.9 ml (range: 30.8 - 134 ml).

### Primary objective #1: treatment-associated toxicity

Adverse events that occurred over the duration of the study are reported in **tables S2B **[see additional file 2: Table S2B] **and S3 **[see additional file 3], together with the initial KPS of the patients and daily dose of Sulfasalazine intake. All patients experienced adverse events (AEs) of grade 1 to 3 (CTCAE V 3.0), with an average of 7.2 ± 2.6 (range 4 - 11, median 7.5). Four (40%) patients developed a grade 4 toxicity, two of whom subsequently developed grade 5 (i.e., lethal) SAEs. The number of adverse events of grade 1 to 3 and the occurrence of adverse events of grade 4 were not related to the dose of sulfasalazine (p = 0.44 and p = 0.17 respectively). Both patients encountering grade 5 adverse events were in the 1.5 g/day dose (p = 0.094).

**Figure 1:**
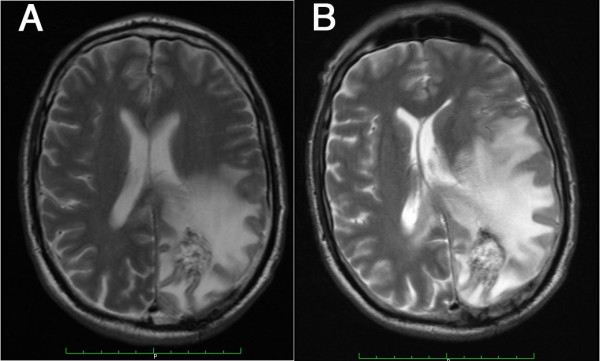
**Sulfaslazine-associated peritumor oedema**. T2 MRI scans obtained at inclusion, prior to Sulfasalazine treatment in patient #4, and 8 days after the initiation of sulfaslaazine treatment (6 g/day). The images demonstrate an increased peritumoral oedema and the developing midline shift. This patient alo developed severe headaches and withdrew from the study.

Of the grade 1-3 AEs, 5 patients developed significant T2 MRI changes, believed to result from an increased peritumoral oedema and accompanied by increased cephalalgia (Figure 1). Proteinuria was encountered in 6 patients during sulfasalazine treatment. Bone marrow toxicity was finally encountered in 4 patients (neutropenia in 2, lymphopenia and thrombopenia in 1 patient each). Four patients also presented reduced serum levels of the anti-epileptic drugs sodium valproate (n = 4), phenytoin (n = 2) and carbamazepine (n = 1). No correlation was observed between sulfasalazine dose and the occurrence of these decreases.

Grade 4 AEs consisted of increased neurological deficits, epilepsy, cognitive dysfunction, altered consciousness, confusion, ataxia and one case of decreased sodium valproate and phenytoin serum levels. Death occurred in this latter patient as the result of inhalation pneumonia, following status epilepticus, after four weeks of Sulfasalazine treatment. Another patient developed a rapidly progressive cognitive deterioration within 5 days of sulfasalazine intake, followed by progressive loss of consciousness and death within a month despite medication withdrawal. Even though the initial KPS of these patients were 40 and 50 respectively at the time of inclusion, these adverse events were considered as probably related to the study drug.

### Primary objective #2: Tumor response

**Figure 2:**
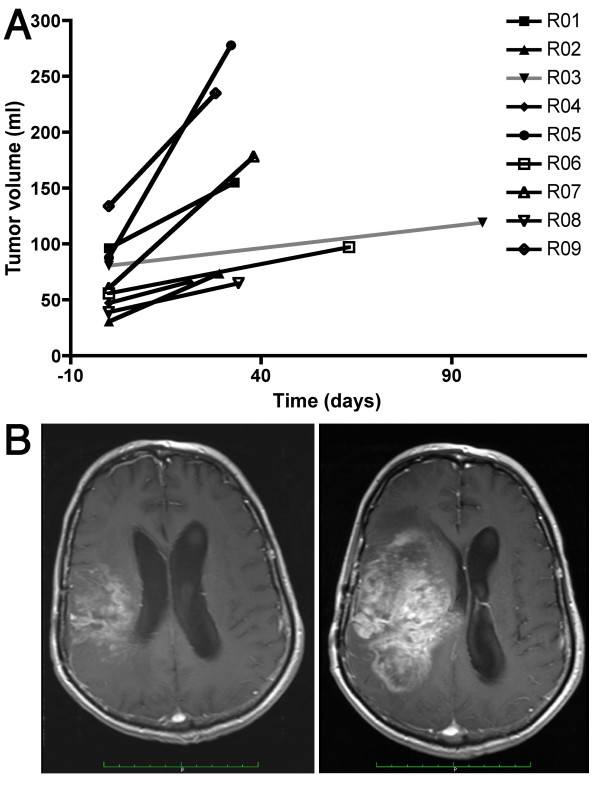
**Tumor growth during Sulfasalazine treatment**. A/Tumor growth between inclusion and sulfasalazine treatment arrest (volumes are provided in ml and were measured by segmentation on gadolinium-enhanced T1 MRI scans); B/Gadolinium-enhanced T1 MRI scans of patient #05 at the time of inclusion and after 32 days of sulfasalzine treatment (6 g/day). The tumor volume has more than tripled over this period.

Only one tumor appeared to be stabilized for two month under sulfasalazine treatment (patient #3), whereas all other lesions progressed. Defining tumoral growth as the ratio between the final and initial tumor volumes, the increase during the treatment duration was on average 2.0 ± 0.7 (range: 1.4 - 3.2) and was highly significant (p < 0.0001). The growth was neither related to the dose of the sulfasalazine treatment (r = 0.45, p = 0.22) nor to treatment duration (r = -0.06, p = 0.88). The tumoral growth rate per day was defined as the difference between the final and the initial volume over the period of sulfasalazine treatment. The growth rate was on average 2.1 ± 1.8 ml/day (range: 0.4 - 5.9 ml/day, Figure 2) and was significantly different from 0 (p = 0.005). The growth rate was not related to the dose (r = 0.26, p = 0.50).

### Secondary objectives: Progression-free and overall survival

**Figure 3:**
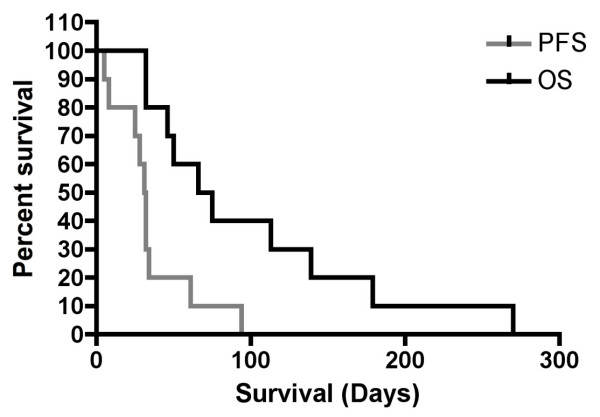
**PFS and overasll survival of ISRCTN45828668**. Kaplan-Meier estimates of the PFS and overall survival of the patients from the time of inclusion in ISRCTN45828668.

The overall survival was defined as the time (days) between the initiation of the sulfasalazine treatment and the death of the patient. Patients survived on average 100 ± 76.9 days (range: 32 - 270 days, median: 70.5 days; Figure 3 and **Table S2B **[see additional file 2]). Treatment was discontinued due to tumor growh (9 patients) and/or side effects (2 patients) after a mean of 35 ± 25.7 days (Progression Free Survival: median: 31.5 days, range: 5-94).

After sulfaslazine discontinuation, one patient was reoperated, and 4 patients (including the latter) received a salvage course of BCNU chemotherapy. The survival time after the end of the sulfasalazine treatment was on average 65.2 ± 55.8 days (range: 0 - 176 days, median: 44.5 days).

## Discussion

The ISRCTN45828668 study was designed to assess the safety and potential efficacy of Sulfasalazine, an IKK and X-cystine transporter inhibitor, for the treatment of recurrent astrocytic malignant gliomas.

As scheduled, an interim analysis of the outcome of the first ten patients included in the study was performed prior to the inclusion of the second half of the planned study population. As described in the results section, this interim analysis revealed a high incidence of serious side effects as well as a lack of efficacy.

Several side effects were observed in our patients, two of which are particularly relevant in the setting of neurosurgical patients. Five patients developed T2 MRI changes characterized by an increase in the apparent peritumoral edema. Sulfasalazine was previously reported to induce encephalopathies and T2 MRI hyperintesities in rheumatologic [23,24], or Crohn's disease patients [25] and to cause intracranial hypertension [26]. It was also reported to aggravate experimental auto-immune encephalomyelitis, a phenomenon thought to result from immune effects and hypersensitivity reactions to the drug [27]. None of our patients, however, presented any rash or other sign of sulfasalzine hypersensitivity. We thus suspect that sulfasalzine more directly increased the permeability of (peri)tumoral vessels in our patients. Such an effect is however unlikely to result from sulfasalazine-mediated NF-kappaB inhibition, as this transcription factor is known to contribute to the vascular leakage in response to LPS and various cytokines [28]. More probably, inhibition of the x(c)-cystine/glutamate antiport could reduce intracellular gluthatione levels in endothelial cells and result in blood-brain barrier dysfunction [29]. Whatever the exact mechanism, and although tumor progression may also have contributed to this effect, sulfasalazine treatment induced a significant brain edema in 50% of our recurrent glioma patients, which likely contributed to worsening of their headaches and neurological condition.

Sulfasalzine treatment correlated with decreased serum levels of anti-epileptic drugs in 5 patients, namely sodium valproate (n = 5), phenytoin (n = 3) and carbamazepine (n = 1). The cause of this side effect remains unclear, as we did not find evidence of any correlation between sulfasalazine dosage and AED level decrease. Seizure activity nevertheless increased in two of these patients, leading to a worsening of their clinical condition and to the death (by inhalation pneumonia) of one of them.

Tumor growth was at best unaffected in our patients following sulfasalazine treatment, in contrast to previous reports of tumor growth inhibition in preclinical in vivo models of human malignant gliomas [2,15]. Several factors might contribute to this discrepancy. First, the small number and large tumor burden of the patients analyzed in this study may have missed or masked a limited drug efficacy. Second, of the 8 tumors for which MGMT promoter analysis could be performed, 7 (87.5%) were unmethylated, a known factor of overall tumor resistance against alkylating agent therapy [30]. Third, the overall pharmacokinetics of sulfasalazine might differ between mice and humans, and different routes of administration were used in preclinical and clinical experiments (intraperitoneal injection in mice, oral use in humans). Fourth, Sulfasalazine targets only one of several pathways to NF-kappaB activation, which may result in malignant gliomas escaping treatment effect by utilizing accessory NF-kappaB pathways. Interestingly, an immunohistochemistry to p65 was performed on the tumor of the patient who was re-operated after sulfasalazine cessation, and showed abundant cytoplasmic and nuclear NF-kappaB reactivity. These highly resistant and heterogeneous tumors could thus rapidly develop mechanisms of resistance to sulfasalazine and/or IKK-independent routes of NF-kappaB activation. Accordingly, PFS during sulfasalazine treatment remained marginal. In addition, differences in NF-kappaB-related pathways may exist between malignant glioma cell lines, primary cultures of malignant glioma, and malignant gliomas specimens [31]. Fifth, Sulfasalazine is known to induce a release of adenosine from several cell types [32], and adenosine is a know mitogen for glial cells [33].

Overall, we did not evidence any objective clinical response to sulfasalazine in this study, and on the contrary, observed some very fast tumor growths despite sulfasalazine treatment and the occurrence of serious adverse events. The death of two patients during or shortly after sulfasalazine treatment raises significant concerns about the safety of this drug for the treatment of human malignant gliomas, even if continued tumor progression in these already debilitated patients (KPS of 40 and 50, respectively) may have contributed to their deaths. In agreement with the independent review committee of the study, these serious adverse events and apparent lack of therapeutic benefit led to the early termination of ISRCTN445828668.

## Conclusion

In conclusion, we strongly oppose the opinion, advocated by several physician and patient support web sites and preclinical reports, that sulfasalazine should be used routinely for the treatment of glioblastoma patients. We also recommend to exert extreme care in the surveillance of glioma patients included or to be included in other controlled clinical trials that use this drug.

However, the strong preclinical evidence of NF-kappaB in the growth and resistance of malignant gliomas remains unaltered by this study, which only raises questions regarding the value of Sulfasalazine as a clinically useful NF-kappaB inhibitor. We thus encourage the development of additional clinical trials that target this transcription factor, using other more specific and/or more potent pharmacological inhibitors of NF-kappaB that are currently being developed (reviewed in [34]).

## Competing interests

The authors declare that they have no competing interests.

## Authors' contributions

PR participated to the design of the study, performed image and data analysis and clinical assessments, and drafted the manuscript, DM participated to the patient recruitment and to the design of the study, MNK participated to image and data collection and analysis, and participated to the discussion of the manuscript, MA participated to data analysis and discussion of the results, MD performed the histological analyses and NF-kappaB immunostaining, AA participated to the design of the study and, together with SV, performed the statistical analyses and participated to the redaction of the manuscript, SC performed the MGMT methylation assays, MB participated to the analysis of the data, discussion of the results and redaction of the manuscript and VB participated to the design of the study and to the discussion of the results as well as to the redaction of the manuscript. All authors read and approved the final manuscript.

## Aknowledgements

The authors wish to thank Dr F. Collignon and J. Lenelle for participating to the patient recruitment. PAR and MA are respectively a Research Associate a 'Télévie' Research Assistant of the National Fund for Scientific Research of Belgium (FNRS), This work was supported by grants from the Leon Frederic Fund (study sponsor), the Belgian Foundation against Cancer, the University Hospital of Liège (FIRS) and the FNRS of Belgium.

## Pre-publication history

The pre-publication history for this paper can be accessed here:

http://www.biomedcentral.com/1471-2407/9/372/prepub

## Supplementary Material

Additional file 1 **Table S1**. inclusion and exclusion criteria of ISRCTN45828668. Click here for file

Additional file 2 **Table S2 A&B**. Characteristics and evolution of patients included in ISRCTN45828668. Click here for file

Additional file 3 **Table S3**. Summary of the adverse events encountered during ISRCTN45828668, classified according to CTCAE V.3.0. Click here for file
