# Supplementary material for: Early termination of ISRCTN45828668, a phase 1/2 prospective, randomized study of Sulfasalazine for the treatment of progressing malignant gliomas in adults
Source: BMC Cancer. 2009 Oct 19;9:372. doi: 10.1186/1471-2407-9-372 (PMC2771045; doi:10.1186/1471-2407-9-372)
Supplement: Additional file 1 — Table S1. inclusion and exclusion criteria of ISRCTN45828668. [file 1471-2407-9-372-S1.PDF]

Table S1: inclusion and exclusion criteria of ISRCTN45828668

**Inclusion criteria**
Age > 18 year
Recurrent or progressive anaplastic astrocytoma or glioblastoma multiforme (WHO grade 3 and 4 astrocytic gliomas), based on MacDonald's criteria {MacDonald 1990}.
Prior treatment consisting of surgery, standard radiation therapy and a first line of conventional chemotherapy (e.g., Temozolomide, CCNU or BCNU).
Written informed consent obtained from the patient.
Histopathological review of previous surgical sample(s) to confirm the diagnostic of grade 3 or 4 astrocytic glioma (*cf.* standard treatment).
Life expectancy ≥ 2 months.
**Exclusion criteria**
Anaplastic oligodendroglioma (WHO grade 3)
Allergy to sulfa drugs
Porphyria
G-6-PD deficiency
Mercaptopurine treatment
Psychiatric disorder deemed incompatible with compliance to the study
Creatinine > 15 mg/l
TGO> 200 UI/l
Amylase > 150 UI/l
Pregnancy or breast feeding
Other experimental medication received within last 30 days (and at least five drug half-lives)
