# Supplementary material for: Early termination of ISRCTN45828668, a phase 1/2 prospective, randomized study of Sulfasalazine for the treatment of progressing malignant gliomas in adults
Source: BMC Cancer. 2009 Oct 19;9:372. doi: 10.1186/1471-2407-9-372 (PMC2771045; doi:10.1186/1471-2407-9-372)
Supplement: Additional file 2 — Table S2 A&B. Characteristics and evolution of patients included in ISRCTN45828668. [file 1471-2407-9-372-S2.PDF]

**A**	**Age**	**KPS**	**Sex**	**Histology**	**MGMT Promoter**	**Localization**	**Prior treatments**	**Survival prior to inclusion**	**Initial volume**
**R01**	45	40	M	GBM	U	R Temporal	S => XRT => TMZ	502	96.44
**R02**	42	70	M	GBM	U	R Parietal	S => XRT => TMZ	483	30.8
**R03**	41	40	M	GBM	M	L Frontal	S => XRT => TMZ => BCNU	693	80.79
**R04**	55	40	M	GBM	U	L Parietal	S => XRT => TMZ	311	47.21
**R05**	55	50	M	GBM	U	L Frontal	S => XRT => TMZ	367	87.87
**R06**	52	40	M	GBM	U	L Frontal	S => XRT => TMZ	833	55.75
**R07**	61	50	M	GBM	NA	R Fronto-Parietal	S => XRT + TMZ	493	60.59
**R08**	62	60	M	GBM	U	Bifrontal	S => XRT => TMZ	203	39.2
**R09**	32	50	F	AA	NA	L Parietal	S => XRT =>PCV => TMZ	984	134
**R10**	57	50	M	AA	U	Multifocal	S => XRT => TMZ	614	44

**B**	**dose (g/day)**	**PFS (days) + cause of discontinuation**	**AE Grades 1-3**	**AE Grade 4**	**AE Grade 5**	**Treatment after Sulfa**	**OS**
**R01**	1.5	32 (Death/ Tumor Growth)	4	2	1	-	32
**R02**	6	25 (Side Effects/ Tumor Growth)	11			BCNU	139
**R03**	1.5	94 (Tumor Growth)	10			-	270
**R04**	6	8 (Side Effects)	10			-	50
**R05**	6	31(Tumor Growth)	7			-	66
**R06**	4.5	61(Tumor Growth)	8			S + BCNU	179
**R07**	6	34 (Tumor Growth)	4	1		-	45
**R08**	3	32 (Tumor Growth)	5			BCNU	113
**R09**	1.5	28 (Tumor Growth)	8	2		BCNU	75
**R10**	1.5	5 (Side Effects)	5	2	1	-	32

Table S2: Characteristics and evolution of patients included in ISRCTN45828668 (**A)** Demographics. (**B**) Progression free survival (PFS), adverse events (AE) according to the CTCAE v3.0, further salvage treatments and overall survival after incorporation in the study.

Legend: KPS= Karnofsky performance score; U= umethylated, M= methylated, NA= unknown; S= Surgery, XRT= radiation therapy, TMZ= Temozolomide, BCNU= Carmustine, PCV= Procarbazine, Lomustine and Vincristine.
