# Supplementary material for: Early termination of ISRCTN45828668, a phase 1/2 prospective, randomized study of Sulfasalazine for the treatment of progressing malignant gliomas in adults
Source: BMC Cancer. 2009 Oct 19;9:372. doi: 10.1186/1471-2407-9-372 (PMC2771045; doi:10.1186/1471-2407-9-372)
Supplement: Additional file 3 — Table S3. Summary of the adverse events encountered during ISRCTN45828668, classified according to CTCAE V.3.0. [file 1471-2407-9-372-S3.PDF]

CTCAE V3.0 code	Grades 1-2	Grade 3	Grade 4	Grade 5
**Neurological**				
Increased neuro deficit (All subtypes)	7	6	2	
MRI Changes (Leukoencephalopathy-T2 hyperintensity)	3	2		
increased seizure frequency	1	1	1	
Cognitive Dysfunction		1	1	
Depressed level of conciousness				1
Ataxia (static)			1	
Confusion			1	
Dysgueusia (Cranial nerve 7)	1			
Mood alteration (depression)		1		
Mood alteration (Anxiety)	1			
**Blood/Bone Marrow**				
Neutropenia	2			
Thrombocytopenia	1			
Lymphopenia	1			
**Laboratory**				
Proteinuria	6			
Increased SPGT	4			
Increased Amylases	2			
Inflammatory syndrome (increased CRP)	2			
Decreased AED serum levels	4		1	
Hypoalbuminemia	1			
Increased SOGT	1			
Increased GGT	1			
**Constitutional Symptoms**				
Fatigue	3			
**Gastrointestinal**				
nausea	2	1		
Anorexia	1			
Constipation	1			
Diarrhea	1			
**Pulmonary/Upper respiratory**				
epistaxis	1			
Pleural effusion	1			
**Lymphatics**				
Oedema Limb	2			
Oedema Head and Neck	1			
**Pain**				
Headache	3	2		
**Infection**				
Pneumonia	1			1
**Coagulation**				
Decreased Plasma Fibrinogen		1		
**Renal/Genitourinary**				
Urine color change	2			
**TOTAL**	**57**	**15**	**7 (*)**	**2**

Table S3: Summary of the adverse events encountered during ISRCTN45828668, classified according to CTCAE V.3.0 . (*: these AE occurred in a total of 4 patients)
